# Stromelysin-3 over-expression enhances tumourigenesis in MCF-7 and MDA-MB-231 breast cancer cell lines: involvement of the IGF-1 signalling pathway

**DOI:** 10.1186/1471-2407-7-12

**Published:** 2007-01-17

**Authors:** Grit Kasper, Matthias Reule, Miriam Tschirschmann, Niels Dankert, Karen Stout-Weider, Roland Lauster, Evelin Schrock, Detlev Mennerich, Georg N Duda, Kerstin E Lehmann

**Affiliations:** 1 Musculoskeletal Research Center Berlin, Charité – Universitätsmedizin Berlin, Berlin, Germany; 2 Astex Therapeutics, Cambridge, UK; 3 University for Technologies, Berlin, Germany; 4 Institute of Medical Genetics, Charité – Universitätsmedizin Berlin, Berlin, Germany; 5 Institute of Clinical Genetics, Technische Universität, Dresden, Germany; 6 Boehringer Ingelheim Pharma GmbH & CoKG, Biberach, Germany; 7 Center for Cardiovascular Research, Charité – Universitätsmedizin Berlin, Berlin, Germany

## Abstract

**Background:**

Stromelysin-3 (*ST-3*) is over-expressed in the majority of human carcinomas including breast carcinoma. Due to its known effect in promoting tumour formation, but its impeding effect on metastasis, a dual role of ST-3 in tumour progression, depending on the cellular grade of dedifferentiation, was hypothesized.

**Methods:**

The present study was designed to investigate the influence of ST-3 *in vivo *and *in vitro *on the oestrogen-dependent, non-invasive MCF-7 breast carcinoma cell line as well as on the oestrogen-independent, invasive MDA-MB-231 breast carcinoma cell line. Therefore an orthotopic human xenograft tumour model in nude mice, as well as a 3D matrigel cell culture system, were employed.

**Results:**

Using both *in vitro *and *in vivo *techniques, we have demonstrated that over-expression of ST-3 in MCF-7 and MDA-MB-231 cells leads to both increased cell numbers and tumour volumes. This observation was dependent upon the presence of growth factors. In particular, the enhanced proliferative capacity was in MCF-7/ST-3 completely and in MDA-MB-231/ST-3 cells partially dependent on the IGF-1 signalling pathway. Microarray analysis of *ST-3 *over-expressing cells revealed that in addition to cell proliferation, further biological processes seemed to be affected, such as cell motility and stress response. The MAPK-pathway as well as the Wnt and PI3-kinase pathways, appear to also play a potential role. Furthermore, we have demonstrated that breast cancer cell lines of different differentiation status, as well as the non-tumourigenic cell line MCF-10A, have a comparable capability to induce endogenous *ST-3 *expression in fibroblasts.

**Conclusion:**

These data reveal that ST-3 is capable of enhancing tumourigenesis in highly differentiated "early stage" breast cancer cell lines as well as in further progressed breast cancer cell lines that have already undergone epithelial-mesenchymal transition. We propose that *ST-3 *induction in tumour fibroblasts leads to the stimulation of the IGF-1R pathway in carcinoma cells, thus enhancing their proliferative capacity. In addition, further different cellular processes seem to be activated by ST-3, possibly accounting for the dual role of ST-3 in tumour progression and metastasis.

## Background

The human protein family of matrix metalloproteinases (MMPs) includes at least 25 members that are believed to play a role in the various stages of tumourigenesis [1,2]. Most of these zinc-dependent endopeptidases cleave more than one extracellular matrix (ECM) component and each ECM component is generally cleaved by more than one MMP. Aside from matrix degradation, MMPs play an important role in releasing growth factors, such as TGF-β or VEGF, or other biologically active factors [3,4]. The effect of over-expression or inhibition of MMPs in tumours might be complex, as these proteases are able to promote and impede tumour relevant processes such as proliferation, apoptosis, angiogenesis and metastasis [5].

Stromelysin-3 (*ST-3, MMP-11*) was originally identified as a breast cancer associated gene, which is over-expressed in more than 90% of invasive breast carcinomas [6]. Recent studies have shown that *ST-3 *over-expression is found in most human carcinomas [7]. Expression of the gene has also been detected in fibroblasts at the invasion front of tumours in close proximity to carcinoma cells [6]. Furthermore, it has been shown that *ST-3 *expression is induced in fibroblasts by co-culturing with breast carcinoma cells [8,9]. Although ST-3 was discovered more than a decade ago and assigned to the family of MMPs due to sequence homologies, no ECM component has yet been identified that is cleaved by full length human ST-3 [10]. However, ST-3 has been found to cleave IGFBP-1, thereby releasing IGF-1 [11]. IGF-1 is known to stimulate proliferation, enhancing survival and migration of cancer cells [12]. Despite a limited knowledge of ST-3 substrates and mode of action, an *in vitro *study has demonstrated that *ST-3 *over-expression in MCF-7 cells is capable of increasing cell survival under serum starvation conditions [13]. However, the mechanism of *ST-3 *activation during tumourigenesis remains unclear. Various *in vivo *studies reveal a ST-3 specific function in tumourigenesis e.g. ST-3 enhanced tumour take of MCF-7 cells in nude mice when injected subcutaneously [14]. Other studies, performed in an *ST-3 *knock out mouse model and in human xenograft models of *ST-3 *over-expressing MCF-7 cells demonstrate that the activation of *ST-3 *leads to a lower percentage of apoptotic cells in the induced tumours [15,16]. According to these results, the generation of MMTV-ras transgenic mice in an *ST-3 *deficient genotype results in the development of less and smaller primary tumours [17]. Interestingly, these mice developed more lung metastases. The question has therefore arisen, whether *ST-3 *over-expression may cause different functional effects in different stages of tumourigenesis and thereby play a dual role [17,18], or whether compensation by other MMP family members could account for the observed effect. Indeed, it has been shown that during involution, *ST-1 *and *ST-2 *is up-regulated in matrilysin knock out mice, and that matrilysin and *ST-2 *expression is elevated in *ST-1 *deficient mice [19].

In the present study, the influence of ST-3 on cell lines of different stages of breast cancer progression is investigated. To this end, the induction of endogenous *ST-3 *expression and the effect of *ST-3 *over-expression on the non-invasive cell line MCF-7 as well as on the highly invasive and dedifferentiated cell line MDA-MB-231 is analysed both *in vitro *and *in vivo*. Furthermore, the impact of *ST-3 *over-expression on the gene expression pattern of breast cancer cells was determined.

## Methods

### Cell culture

Primary fibroblasts were isolated from human breast tumours (kindly provided by Dr. Têtu, Centre Hospitalier Universitaire de Quebec, Canada)[8]. MDA-MB-231 cells and primary breast tumour fibroblasts were maintained in DMEM/F12. MCF-7 cells were maintained in RPMI w/o phenol red (Gibco, Germany) supplemented with 0.002 U/ml insulin and 0.1 nM Estradiol (E_2_). For MDA-MB-468, RPMI 1640 (Gibco, Germany) was used. These media contained 10% FCS. MCF-10A cells were maintained in DMEM/F12 (Gibco, Germany) with 5% heat inactivated horse serum, 20 ng/ml EGF, 100 ng/ml cholera toxin, 0.002 U/ml insulin and 500 ng/ml hydrocortisone. For 3D matrigel cultures, 96 well plates were pre-coated with matrigel (BD Bioscience, 10 mg/ml). 75 μl of a cell/matrigel suspension containing 1 × 10^3 ^cells/μl were added to each well. For co-cultures, 1 × 10^4 ^fibroblasts were seeded on cover slips. When the cells reached confluence, epithelial cells were seeded on top. At day three, co-cultures were incubated with 10 μg/ml Brefeldin A for 5 hours and investigated by immunocytochemistry.

### Immunocytochemistry/Immunohistochemistry

Immunocytochemistry was carried out as described previously [20]. Briefly, cells grown on cover slips were washed with PBS, fixed with 4% PFA and permeabilized in 0.2% Triton-X100/PBS. Cover slips were incubated with the primary and, after washing, with the secondary antibodies for 20 min at RT. For immunohistochemical staining, paraffin sections were dewaxed and dehydrated by graded ethanol. After blocking, sections were incubated with the primary and secondary antibodies. As primary antibodies goat α-(human vimentin) (1:30, Santa Cruz, Germany), rabbit α-(human cytokeratin) (1:100, Novocastra, Newcastle) and mouse α-(human ST-3) (1:50, Clone SL3.05, Neomarkers, CA) were used. Secondary antibodies were donkey-α-(mouse IgG) Rhodamin Red (1:100, Dianova, Germany) and donkey α-(goat IgG)Cy5 (1:400, Dianova, Germany). Stainings were performed as triple stainings on the same section. Samples were examined using a Leica TCS SL confocal microscope (Leica, Germany).

### Generation of ST-3 over-expressing clones

*ST-3 *cDNA was amplified by performing a PCR and cloning into the mammalian expression vector pcDNA3.1V5HisTOPO (Invitrogen, Karlsruhe, Germany). Expression of ST-3 protein was confirmed by *in vitro *transcription/translation in reticulocyte lysate (Promega). The *ST-3 *or empty expression plasmid was transfected into MCF-7 and MDA-MB-231 cells. 6 μl Fugene (Boehringer, Mannheim, Germany) was mixed with 196 μl Optimem (Gibco, Karlsruhe, Germany) and incubated for 5 min at room temperature. After supplementation with 2 μg DNA and a further incubation period of 15 min, this mixture was applied to a 60% confluent 6-well plate. On the following day, transfected cells were seeded at a density of 4000 cells/cm^2 ^and selected by supplementation of media with G418 (750 μg/ml for MDA-MB-231 and 600 μg/ml for MCF-7). Transfection efficiencies were determined using a β-Gal plasmid and were in the range of 10–20%. Three independently derived and randomly chosen cell clones were isolated.

### Western blotting

Cell culture supernatant containing 0.1% FCS was collected after 48 hours of culture and concentrated 20 times by Biomax 5 k filter (Fa. Millipore, MA). Western blotting was carried out according to manufacturer's instructions utilizing the Novex gel system (Invitrogen, Germany) and 4–12% gradient SDS-PAGE. 12 μl of the concentrated supernatants were applied and equal protein loading was confirmed by Ponceau staining after transfer onto nitrocellulose membranes. Immunoreactive proteins were detected by secondary antibody binding (donkey α-(mouse IgG)) conjugated to horseradish peroxidase, (1:50.000, Amersham Bioscience, Germany) followed by incubation with ECL-Plus (0.1 ml/cm^2^, Amersham Biosciences, Germany). The primary antibody, mouse anti-ST-3 (Clone SL3.05, Neomarkers, CA) was used at a dilution of 1:300.

### Proliferation assays

Cells of either three ST-3 or three mock clones were detached by trypsin, counted and mixed in equal parts. For each well, a total number of 4.5 × 10^4 ^cells (i.e. 1.5 × 10^4 ^cells per clone) were mixed with 75 μl matrigel and applied to matrigel coated 96-well plates. Gels were allowed to solidify for 30 min at 37°C before supplementation with 100 μl of cell culture media. After 7 days of 3D culture, cell numbers were determined by means of a MTS assay (Fa. Promega, Germany). The applicability of the MTS test was determined beforehand by confirming the linearity of measurement of known cell numbers in 3D cultures (data not shown). All assays were conducted at least 3 times with triplicates.

### *In vivo *tumourigenesis assay

8–12 week-old female immunocompromised NMRI nude/nude mice were obtained from Harlan Winkelmann (Borchem, Germany). 2 × 10^6 ^cells consisting of equal parts of either three *ST-3 *or mock clones were mixed with 50 μl matrigel and injected into the mammary fat pad. Growth factor depleted matrigel was prepared as described by Taub *et al *[21]. Tumour take and tumour growth was determined by the measurement of tumour burdens estimated from calliper measurements twice a week. The minimum tumour size was defined as 3 × 3 mm. Animals were sacrificed when tumours were larger than 1000 mm^3^. Animals receiving MCF-7 tumour cells were supplemented with 17-beta Estradiol pellets (IRA, FI) that were implanted subcutaneously one week in advance. Animals were sacrificed according to the guidelines of the "Deutsches Tierschutzgesetz".

### Microarray gene expression analysis

Cells were grown for 3 days in 3D matrigel cultures as described above. RNA-isolation was carried out using TriFast (peqLab, Erlangen, Germany). The integrity of resuspended total RNA was measured on a Bioanalyzer gel (Agilent Technologies). For the microarray experiment, 500 ng of total RNA was reverse transcribed with an oligo-(dT)-T7 promoter primer and Moloney murine leukemia virus-reverse transcriptase (MMLV-RT) to synthesize first and second strands of cDNA. Fluorescent antisense cRNA was synthesized with simultaneous incorporation of either cyanine 3-cytidine 5'-triphosphate (3-CTP) or cyanine 5-CTP. The labelling efficiency was verified with a Nanodrop photometer (Kisker). Before hybridisation, 2 μg of each labelled cRNA product were fragmented and mixed with control targets and hybridisation buffer according to the supplier's protocol (Agilent Technologies). Hybridisations were done overnight for ~19 h at 60°C and arrays were subsequently washed. The scanning resolution was 5-μm using a DNA microarray laser scanner (Agilent). Experiments were then repeated, performing a colour swap dye reversal. Features were extracted with an image analysis tool (Agilent Technologies, version A 6.1.1) using default settings. Data analysis was conducted on the Rosetta Inpharmatics Platform Resolver Build version 4.0. Expression patterns were identified by stringent data analysis using anticorrelation of the dye reversal ratio profiles and a 2-fold expression cut-off. Combining the first and the second criteria of analysis, data points were filtered out with a low *p *value (*p *< 0.01). To conduct functional categorizing, all genes with a minimum of two-fold absolute changes were submitted to the Database for Annotation, Visualization and Integrated Discovery (DAVID[22]). Pathway mapping analysis for these genes was done by BioRag (Bioresource for array genes [23]).

### Statistical analysis

For all statistical analyses, a significance level of p ≤ 0.05 was employed. To test differences in means of cell numbers and tumour volumes, a two sided T-test was used. Differences of tumour take curves were evaluated by means of a log rank test. To conduct these statistical analyses, the software package SPSS 12.0 was employed.

## Results

### Induction of ST-3 expression in co-cultures of breast epithelial cells and fibroblasts

**Figure 1:**
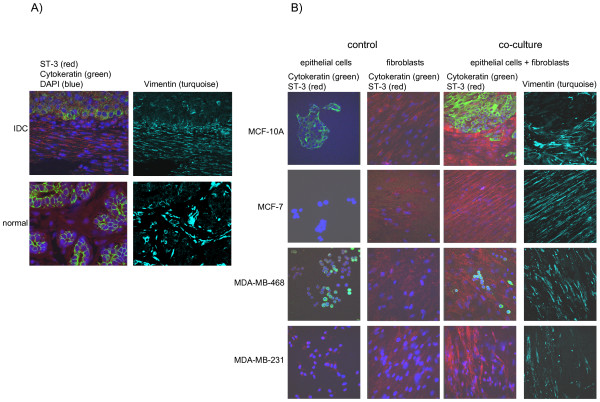
**Induction of *ST-3 *expression in breast cancer fibroblast by breast epithelial cells**. A) Invasive ductal carcinoma (IDC) and normal breast tissue as well as B) co-cultures with different breast cell lines were stained by immunohistochemistry and immunocytochemistry, respectively. Similar expression patterns are observed in co-cultures and IDC tissue section. A triple staining of ST-3 (red), vimentin (turquoise) and E-cadherin (green) was performed and analysed by confocal microscopy. Nuclei were counterstained by DAPI. Original magnification: 630×.

The expression of *ST-3 *in fibroblasts of breast cancer tissue was confirmed by co-staining with marker proteins for fibroblasts and epithelial cells (Figure 1A). Epithelial cells of both normal breast tissue and of IDC showed a low *ST-3 *expression. Also fibroblasts surrounding normal ducts expressed low amounts of ST-3, indicating a low level basal expression of the protein. To determine *in vitro*, whether *ST-3 *expression in fibroblasts is induced by epithelial cells of different tumourigenic and invasive potential, the breast cancer cell lines MCF-7, MDA-MB-468 and MDA-MB-231 and the non-tumourigenic, immortalized cell line MCF-10A were co-cultured with primary breast tumour fibroblasts (figure 1B). Fibroblasts were induced to express endogenous *ST-3 *by all four cell lines at similar levels, independently of their tumourigenic or invasive properties. In accordance with the fibroblastoid appearance of MDA-MB-231 cells, they were vimentin positive, cytokeratin negative and showed low *ST-3 *expression (figure 1B and data not shown). The epithelial MCF-7 cells displayed typical characteristics (epithelial morphology, estrogen dependency and lack of caspase 3 activity, data not shown) but did not stain positively for cytokeratin. As the MCF-10A and MDA-MB-468 cells were both positive for this marker, it is possible that the cells might have acquired a mutation in the antigenic sequence not recognized by the anti-cytokeratin antibody used.

### Characterisation of ST-3 over-expressing clones

**Figure 2:**
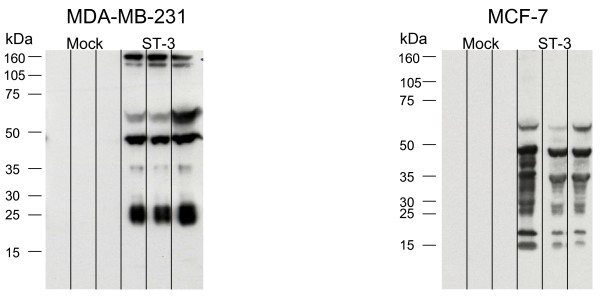
**Characterisation of *ST-3 *over-expressing cell clones**. Conditioned media of *ST-3 *and mock transfected clones was analysed by Western blotting. Proform (~60 kDa), active form (~50 kDa) and degradation products (~35 kDa, ~28 kDa) are detected at similar amounts in *ST-3 *over-expressing clones of the cell lines MDA-MB-231 and MCF-7.

To investigate the functional consequences of such *ST-3 *over-expression, the non-invasive cell line MCF-7, as well as the invasive cell line MDA-MB-231, were stable transfected with *ST-3*. FISH analysis revealed a single copy integration of each of the *ST-3 *and mock transfected clones, as well as their clonality (data not shown). Each separate clone displayed a different site of integration. Western blot analysis proved that the over-expressed ST-3 protein displayed similar molecular weight patterns in MCF-7 and MDA-MB-231 clones, corresponding to the published molecular weight of *ST-3 *gene products (figure 2) [24].

### Functional analysis *in vivo*

**Figure 3:**
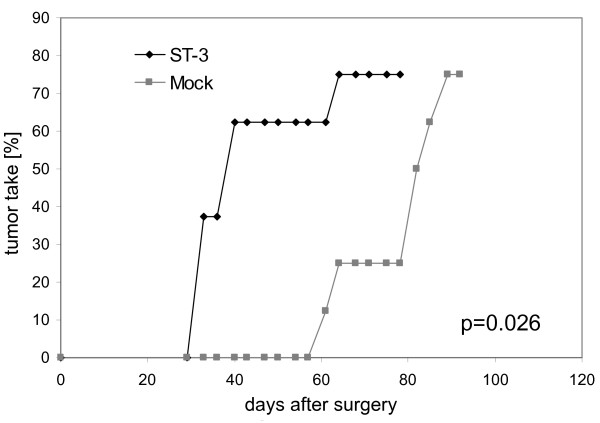
***ST-3 *over-expression increases tumour take of MDA-MB-231 cells *in vivo***. A pool of equal parts of three cell clones (total cell number: 2 × 10^6^) was injected in 50 μl of a 1:1 matrigel/PBS mixture into the mammary fatpad of nude mice. Tumour take was monitored twice a week. Groups included eight animals each. Stars indicate a p-value ≤ 0.05.

**Figure 4:**
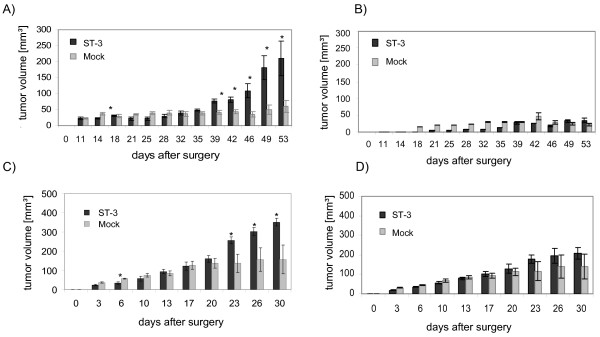
**ST-3 induced increase of tumour volume is dependent on the presence of growth factors *in vivo***. Tumour volume in relation to the day of surgery is shown from A) MDA-MB-231 cell clones delivered with complete matrigel, B) MDA-MB-231 cell clones applied in growth factor depleted matrigel, C) MCF-7 cell clones injected with complete matrigel and D) MCF-7 cells applied with growth factor depleted matrigel. A pool of equal parts of three cell clones (total number: 2 × 10^6^) was injected in a volume of 50 μl of (depleted) matrigel into the mammary fatpad of nude mice. Tumour volume was monitored twice a week. One week prior to MCF-7 cell transplantation, animals received oestrogen pellets subcutanously. Groups included eight animals each. Stars indicate a p-value ≤ 0.05.

The tumourigenic potency of transfected cells was investigated in an orthotopic xenograft model in nude mice. As demonstrated in Figure 3, *ST-3 *over-expression in MDA-MB-231 cells resulted in an elevated tumour take rate (p = 0.026). Mock transfected clones lead to a similar final tumour take rate of 75%, but at a significantly delayed time point. In addition, larger tumour volumes were observed in *ST-3 *over-expressing MDA-MB-231 as well as MCF-7 cells compared to mock transfected control cells in the presence of complete matrigel (Figure 4A, p = 0.043 at day 53; Figure 4C, p = 0.024 at day 30). In contrast, when growth factor depleted matrigel was employed no differences between *ST-3 *and mock transfected cell clones were detected (Figure 4B, p = 0.287 at day 53; Figure 4D, p = 0.373 at day 30). Tumour volume of *ST-3 *transfected and control cells did not become significantly larger until day 39 for MDA-MB-231/ST-3 and day 23 for MCF-7/ST-3 after tumour cell injection. These data indicate that not only the tumour settlement is enhanced by *ST-3 *over-expression, but also the gain in tumour volume is accelerated. This finding becomes obvious when the tumour volume is normalized to the day of occurrence instead of the day of injection, so that the bias resulting from different tumour takes are eliminated. Statistically significant enhancement of tumour volume was achieved on day 18 for MDA-MB-231 clones (p = 0.028 and p = 0.422 for complete and reduced martigel respectively) and on day 11 for MCF-7 clones (p = 0.034 and p = 0.309 for complete and reduced matrigel respectively). These *in vivo *experiments demonstrate that ST-3 causes similar effects in MDA-MB-231 and MCF-7 cells by increasing the tumour take as well as the tumour growth rate.

### Analysis of cell proliferation in *in vitro *3D matrigel cultures

**Figure 5:**
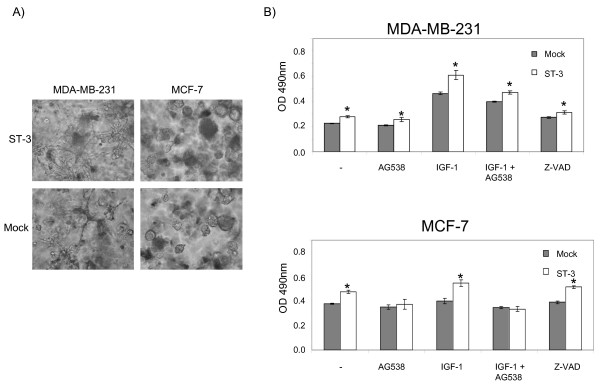
***ST-3 *over-expression results in an increased cell number *in vitro***. A) Cell morphology of representative clones of MDA-MB-231 and MCF-7 cells transfected with *ST-3 *expression plasmid or empty vector (mock) in 3D matrigel cultures. MDA-MB-231 clones show typical invasive structures, whereas MCF-7 cells formed rounded spheroids. Clusters appeared to be slightly larger in *ST-3 *transfected clones. Morphology seems not to be influenced by *ST-3 *over-expression. Original magnification: 20×. B) OD 490 nm, which is proportional to cell numbers is shown for MDA-MB-231 and MCF-7 cell clones. A pool of equal parts of three clones was grown for 7 days in matrigel cultures with media containing 1% FCS with and without supplementation of: AG-538 [100 μM], IGF-1 [50 ng/ml] and Z-VAD [50 μM]. Equal seeding for *ST-3 *and mock clones was confirmed at day 0. Stars represent significant differences in the cell number of mock and *ST-3 *transfected cells (p-value ≤ 0.05).

To investigate the effect of ST-3 over-expression *in vitro*, a 3D matrigel model was established. The morphology of *ST-3 *over-expressing clones and their corresponding control clones from MDA-MB-231 and MCF-7 was similar. In general, cell clusters of *ST-3 *expressing cells appeared to be larger in size (Figure 5A). To determine cell numbers, a MTS test was employed and the linearity of measurement of known cell numbers and OD_490 nm _was confirmed in the 3D cultures (data not shown). Indeed, *ST-3 *over-expressing cell clones showed a significantly higher cell number compared with mock transfected control cells (Figure 5B, MCF-7: 20.2% increase, MDA-MB-231: 23.2% increase). This effect was not observed in standard 2D cultures on cell culture dishes (data not shown). The addition of IGF-1 led to a further increase in the MDA-MB-231/ST-3 cell number compared to mock transfected clones. This difference was still present, but reduced by the addition of IGF-1R inhibitor AG-538. In contrast, the addition of AG-538 to MCF7/ST-3 and control cells, either alone or in the presence of IGF-1, resulted in an equalization of cell numbers in MCF-7/ST-3 and mock cells. These data suggest that the activation of the IGF-1 signalling pathway is required for ST-3 action in MCF-7 cells, whereas additional mechanisms seem to play a role in MDA-MB-231 cells. The addition of apoptosis inhibitor Z-VAD only slightly decreased the effect of ST-3 in MDA-MB-231 clones and had no significant impact on MCF-7 cell clones.

### Analysis of ST-3 influence on gene expression pattern

**Figure 6:**
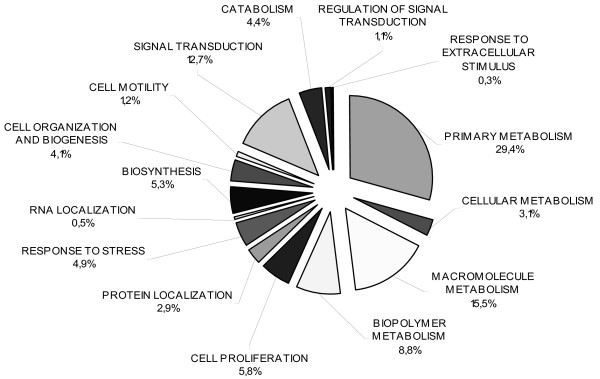
**ST-3 induced changes in expression pattern of MDA-MB-231 cells**. The pie diagram depicts gene distribution based on functional categories. Genes that were differentially expressed between *ST-3 *and mock clones at a minimum of twofold were included in the analysis.

To investigate potential genes and pathways involved in an ST-3 response apart from IGF-1R signalling, transcriptional profiling of *ST-3 *over-expressing MDA-MB-231 cells in comparison to mock transfected cells was employed. Of the total of 1190 significantly regulated genes the overall number of genes induced by ST-3 was larger than the number of genes that showed a down regulation (911 versus 279). In all, 708 differentially expressed genes could be clustered according to their involvement in biological processes by gene ontology analysis resulting in 15 functional categories (Figure 6). Fourteen of these categories, including signal transduction, cell proliferation, cell motility, and regulators of cell metabolism revealed significant changes in expression (p < 0.05, category "response to extracellular stimulus": p = 0.06; Figure 6). Examining the expression of MMP genes revealed an up-regulation of MMP-28 in *ST-3 *over-expressing cells (NM_032950, 2.9 fold). Up-regulation of the *ST-3 *gene was not been detected at the mRNA level. Since the enhanced protein production was clearly shown by Western blotting (Figure 2), this was probably due to technical reasons. Pathway mapping analyses showed the following signalling pathways that were mainly affected: the MAPK-signalling pathway, pathways involved in cell cycle progression, Wnt-pathway, and the phosphatidylinositol signalling system (Table 1). The expression of small GTPases of the Rho family, RhoC and cdc42 were all up-regulated (2.4 and 5.7 fold) by ST-3 as well as the adapter protein SHC3 (2.2 fold), which has been shown to be involved in PI3-kinase activation promoting cell survival [25]. Transcripts coding for cell cycle regulators required for G1/S transition, such as cyclin dependent kinase 6 and 4 (CDK6, CDK4), CDK1 (also known as cell division cycle 2, CDC2) and CyclinB (CCNB1) were up-regulated, indicating a positive role for ST-3 during cell cycle progression. A number of components of the MAPK signalling pathway were also up-regulated (MAP4K2, MAP4K1, MAP2K4) as well as PDGFRB (but not the IGF-R1), which activates the MAPK- and the Akt pathways indicating a role for growth factor mediated cellular response. However, the MAPK pathway downstream transcription factor AP-1 subunit jun (here v-jun sarcoma virus 17 oncogene homologue, AC: BC002646) and MAP3K2 were down regulated whereas a negative regulator of cell cycle p27KIP1 (CDKN1B, cyclin dependent kinase inhibitor1B) was up-regulated. ST-3 was also found to up-regulate PKN2 (protein kinaseN2, also known as PRK2) and the PI3-kinase catalytic subunit p110 (PIK3CA). The latter is known to promote cell proliferation via downstream activation of p70S6-kinase [26]. PI3-kinase also leads to cell motility (via Rac1 activation) and cell survival (via Akt activation). Furthermore, the anti-apoptotic Bcl-2, which was up-regulated 2.4 fold, points towards a role for ST-3 in cell survival processes. PKN2 was recently shown to be part of a ternary Rho/PDK1 protein complex enhancing Akt activation in a PI3-kinase dependent manner [27]. Components of the Wnt signalling pathway, such as FZD7 (frizzled homologue7) and its downstream target DVL3 (dishevelled, dsh) were up-regulated. These genes are known to be involved in tumour cell proliferation and invasion via activation of the transcription factor TCF/LEF1. However, CTBP-1, an inhibitor of this transcription factor, was up-regulated. These results indicate that *ST-3 *activation induces synthesis of intracellular signalling components characteristic for downstream activation in response to growth factor receptor activation, promoting cell cycle progression and migration. Interestingly, no transcripts for soluble growth factors (e.g. IGF) were induced by *ST-3 *over-expression.

## Discussion

It has been shown in the present study that *ST-3 *induction by an immortalized, non-tumourigenic cell line is similar to the induction by tumourigenic breast cancer cell lines. The stimulation of *ST-3 *expression in breast cancer fibroblasts by different breast tumour cell lines was recently described on mRNA and protein level and is in line with our results [8,9]. Cell-cell contacts seem to be beneficial, but not essential to induce *ST-3 *expression, since *ST-3 *was induced in direct co-cultures and to a lower extent in indirect co-cultures [8]. PKC seems to be involved in the induction of *ST-3 *expression in normal pulmonary fibroblasts by non-small cell lung cancer cells [28]. The observation that non-tumourigenic cells are also able to induce *ST-3 *expression might indicate that the association of *ST-3 *expression and invasive properties of carcinomas is a consequence of invasive epithelial cells coming into contact with fibroblasts rather than being the cause of invasion. The observation, both by our group as well as others, that *ST-3 *over-expression does not lead to an enhanced invasiveness of cells (data not shown and [14]) provides further strength to this hypothesis. Normal as well as tumor derived breast epithelial cells expressed low amounts of *ST-3*. An epithelial expression of *ST-3 *was already described in gastric and pancreatic carcinoma [29,30] and seems to also occur in breast tissue.

ST-3 significantly enhanced tumour take as well as tumour growth rates. Thus, the results indicate that ST-3 is not only involved in tumour formation, as it was postulated by previous studies [14], but additionally in tumour progression. This was true for MCF-7, the breast cancer cell line also used in former *in vivo *experiments [14,31], and for MDA-MB-231 cells. MCF-7 is a rather well differentiated tumour cell line, reflected by its oestrogen-dependent growth. This cell line is non-invasive with characteristics of epithelial cells (e.g. E-cadherin expression and a lack of *ST-3 *expression). In contrast, MDA-MB-231 is a tumour cell line that has undergone epithelial-mesenchymal transition and thus acquired fibroblastoid characteristics (e.g. morphology, invasiveness, lack of E-Cadherin and expression of low amounts of *ST-3*). MDA-MB-231 cell growth is independent of the presence of oestrogen. Thus, ST-3 seems to have similar effects on breast cancer cells derived from more differentiated tumours and on cells from more aggressive and dedifferentiated lesions.

The described *in vitro *results indicate that *ST-3 *over-expression leads to an accelerated cell proliferation. Indeed, ST-3 protein expression has been found to be up-regulated in human invasive breast carcinomas and in co-expression to clinically-used proliferation markers [32]. Since ST-3 has been shown to be capable of releasing IGF-1 bound to IGFBP-1, thereby stimulating proliferation of BAF/3 cells [11], this signalling pathway was investigated in the present study. The IGF-1 signalling cascade seemed to be the only or predominant pathway responsible for the ST-3 effect in the MCF-7 cell line, possibly via the ERK/MAPK pathway. It was described that *ST-3 *over-expression in MCF-7 cells results in the activation of p42/p44 MAP kinase [13]. In contrast, additional pathways seem to be involved in MDA-MB-231 cells. In this respect, it is notable that there seems to be cross-talk between IGF-1 and the estrogen receptor [33].

Transcriptional profiling revealed that cancer related processes such as cell proliferation, cell motility and cell metabolism are affected by ST-3 over-expression. Most notably, cell proliferation and cell cycle progression seem to be influenced, an observation that has been confirmed by our *in vitro *model. ST-3 leads to an up-regulation of the small GTPases of the Rho family, RhoC and cdc42, which have an important role in cancer progression, including proliferation, invasion, and metastasis of breast tumours [34]. Cdc42 and RhoC are induced in response to integrin activation suggesting a role for ST-3 in mediating cell-matrix adhesion, a prerequisite for cell migration. Rho GTPases also play a role in the induction of MMP expression [35-37] and, conversely, blocking Rho function can inhibit transcription of MMPs [38]. Whether this is also true for ST-3 activated Rho GTPases still needs to be proven. Interestingly, MMP-28 expression was induced in *ST-3 *overexpressing cells. This MMP has been described to be involved in EMT in lung carcinoma cells [39] and could additionally contribute to the observed effect.

However, no induction of gene expression of growth factors could be detected in the presence of ST-3, suggesting that ST-3 might not be involved in *de novo *growth factor synthesis, which is crucial for autocrine activation of signalling pathways as a characteristic feature for tumour cells [40]. Moreover, ST-3 seems to be involved in the release and activation of soluble growth factors bound by ECM components. This hypothesis is also supported by our *in vivo *data and the finding that ST-3 can activate extracellular IGF bound to IGFBP-1, which strengthened its definition as a MMP [11]. Thus, our results suggest that ST-3 enhances rather than initiates tumourigenesis by either activating ECM-bound signaling components or at least by synergistic effects with ECM containing growth factors such as IGF-1. Our gene expression data also support a role in *ST-3 *activation of Wnt-signalling components, such as FZD7 and DVL3, which is in line with the activation of the Wnt pathway by ST-1/MMP-3 via E-cadherin cleavage, resulting in EMT and thus enhanced invasive properties of mammary tumour cells [41].

## Conclusion

This study shows that ST-3 expression in fibroblasts is induced not only by epithelial cells of tumour origin, but also by non-transformed cells. Results from an orthotopic *in vivo *model as well as from *in vitro *3D assays have demonstrated that ST-3 leads to an enhanced tumourigenic potency in cell lines of different dedifferentiation status. The mechanisms by which ST-3 exerts its function seem to involve activation of IGF-1 signalling and potentially MAPK and PI3-kinase pathways. Clearly, further studies are needed to confirm and investigate the impact of ST-3 on these pathways, but our data provide some novel clues for the mechanisms of ST-3 activation in breast cancer cells.

## Abbreviations

ST-3: Stromelysin-3, MMP: matrix metalloprotease, IGFBP: insulin like growth factor binding protein, ECM: extracellular matrix, EMT: epithelial mesenchymal transition, FISH: Fluorescence *in situ *hybridisation

## Competing interests

The author(s) declare that they have no competing interests.

## Authors' contributions

GK: participated in study and experiment design, carried out co-cultures, Western blots and immunocytochemistry, generated stable transfected clones, established *in vitro *3D assay, drafted the manuscript

MR: designed, performed and analysed animal experiments

MT: designed, performed and analysed microarray analysis

ND: performed and analysed in vitro cell culture assays

KSW: participated in characterization of stable clones and helped to draft the manuscript

RL: participated in microarray analysis

ES: participated in characterization of stable clones and revised manuscript critically

DM: carried out cloning, participated in immunohistochemistry and discussion of data

GND:participated in experimental design and help to draft the manuscript

KEL: particitpated in confocal microscopy and design of study

All authors read and approved the final manuscript.

## Pre-publication history

The pre-publication history for this paper can be accessed here:



## Figures and Tables

**Table 1:** Pathways affected by *ST-3 *over-expression in MDA-MB-231*

**pathway name**	**accession**	**gene name**	**fold change**	**pathway name**	**accession**	**gene name**	**fold change**
*MAPK signalling pathway*				*Wnt signalling pathway*			
	NM_004630	MAP4K2	3.7		NM_005044	PRKX	-2.1
	NM_016151	thousand and one amino acid protein kinase	2.6		NM_004423	DVL3	2.5
	NM_002524	NRAS	2.3		NM_002335	LRP5	-2.0
	AF188611	HSPA5	-2.1		NM_003507	FZD7	2.4
	NM_005044	PRKX	-2.1		BC002646	JUN	-2.2
	M54968	KRAS2	-2.1		NM_002760	PRKY	3.0
	NM_006609	MAP3K2	-2.0		BC011655	CTBP1	7.3
	NM_006256	PKN2	5.3		NM_002731	PRKACB	2.7
	BC002646	JUN	-2.2	*Ribosome*			
	NM_007181	MAP4K1	3.4		BF570115	RPLP0	2.7
	NM_001791	CDC42	5.7		AK093246	RPL13	2.5
	NM_000719	CACNA1C	2.8		BC022357	RPL17	2.0
	AL050044	GADD45B	2.3		NM_001022	RPS19	5.5
	BC003623	YWHAZ	6.7		NM_016093	RPL26L1	2.3
	AK124299	PPM1A	2.1		NM_001017	RPS13	9.1
	AF233453	PRKCBP1	2.4	*Apoptosis*			
	NM_003010	MAP2K4	2.6		NM_003844	TNFRSF10A	-2.2
	NM_002760	PRKY	3.0		NM_006218	PIK3CA	2.0
	NM_002731	PRKACB	2.7		NM_000633	BCL2	2.4
	NM_002609	PDGFRB	3.3		U84408	MYD88	3.2
*Cell cycle*					NM_002731	PRKACB	2.7
	BC039904	HDAC4	3.0	*Jak-STAT signalling pathway*			
	BC008026		2.9		NM_001560	IL13RA1	2.9
	NM_005915	MCM6	2.1		NM_006218	PIK3CA	2.0
	NM_004725	BUB3	3.6		BC030949	PTPN11	2.6
	NM_000075	CDK4	2.4		NM_000418	IL4R	2.5
	NM_004219	PTTG1	3.4		BC028068	JAK3	-2.2
	NM_031966	CCNB1	2.0	*Ubiquitin mediated proteolysis*			
	NM_001786	CDC2	2.8		NM_003334	UBE1	-2.1
	AL050044	GADD45B	2.3		NM_181838	UBE2D2	3.1
	NM_004064	CDKN1B	2.4		AB012109	ANAPC10	2.4
	NM_006607	PTTG2	2.6		NM_033632	FBXW7	2.7
	BC027989	CDK6	2.6	*Integrin-mediated cell adhesion*			
* analysed by BioRag					NM_005167	RHOC	2.4
					NM_001791	CDC42	5.7
					NM_016848	SHC3	2.2
